# Application of an Ultrafine Shearing Method for the Extraction of C-Phycocyanin from *Spirulina platensis*

**DOI:** 10.3390/molecules22112023

**Published:** 2017-11-21

**Authors:** Jianfeng Yu

**Affiliations:** 1 Food Engineering & Machinery Group, School of Mechanical Engineering, Jiangnan University, 1800 Lihu Avenue, Wuxi 214122, Jiangsu, China; yujf@jiangnan.edu.cn; Tel.: +86-0510-8591-0581; 2 Jiangsu Key Laboratory of Advanced Food Manufacturing Equipment & Technology, 1800 Lihu Avenue, Wuxi 214122, Jiangsu, China

**Keywords:** *Spirulina platensis*, C-phycocyanin, cell disruption, ultrafine shearing method, extraction

## Abstract

Cell disruption is an important step during the extraction of C-phycocyanin from *Spirulina platensis*. An ultrafine shearing method is introduced and combined with soaking and ultrasonication to disrupt the cell walls of *S. platensis* efficiently and economically. Five kinds of cell disruption method, including soaking, ultrasonication, freezing-thawing, soaking-ultrafine shearing and soaking-ultrafine shearing-ultrasonication were applied to break the cell walls of *S. platensis*. The effectiveness of cell breaking was evaluated based on the yield of the C-phycocyanin. The results show that the maximum C-phycocyanin yield was 9.02%, achieved by the soaking-ultrafine shearing-ultrasonication method, followed by soaking (8.43%), soaking-ultrafine shearing (8.89%), freezing and thawing (8.34%), and soaking-ultrasonication (8.62%). The soaking-ultrafine shearing-ultrasonication method is a novel technique for breaking the cell walls of *S. platensis* for the extraction of C-phycocyanin.

## 1. Introduction

*Spirulina platensis* is a blue-green microalgae that has emerged in the market during recent years [1,2]. Its structure is that of a unicellular algae, with a multicellular cylindrical trichome arrangement. *S. platensis* is rich in bioactive components, including C-phycocyanin (C-PC), chlorophyll, carotenoids and unsaturated fatty acids [3]. *S. platensis* has a particularly high protein content (55–65% of dry weight) [4], and 20% of the dry weight of the cell protein is C-PC [5,6].

C-PC is an important light-harvesting protein. C-PC contains a variety of essential amino acids. The application of C-PC in biomedical and food research has expanded continuously in recent years. C-PC plays an important role in many therapeutic applications, such as anti-cancer, anti-oxidation and improvement of physiological function [7]. Because of its strong fluorescence, C-PC is often used as a biological probe for component detection in biomedical research. 

Producing C-PC from microalgae typically includes its extraction and subsequent purification [8]. Because the cellular shape of *S. platensis* is cylindrical and spirally curved, and its cell wall is extremely rigid, cell disruption of *S. platensis* is notoriously difficult. If the cell disruption method is efficient and effective, the yield of C-PC can be improved considerably, and the extraction time can be shortened. Many cell disruption methods (physical, chemical and enzymatic) have been applied to liberating the intracellular materials (lipids or proteins) from *S. platensis* [9,10]. The chemical cell disruption methods may contaminate downstream products [11]. Physical cell disruption techniques including soaking [12], freezing-thawing [9], bead milling [13], high-pressure homogenization [14,15], ultrasonication [16] and microwaves [17] have previously been evaluated in the literature. Safi et al. [18] evaluated four cell disruption methods, including manual grinding, ultrasonication, chemical treatment and high-pressure homogenization, based on the protein concentration released from five microalgae. Safi et al. [19] investigated and compared the effect of cell disruption methods including ultrasonication, chemical hydrolysis, high-pressure homogenization and bead milling on the diffusion of Chlorella vulgaris proteins and pigments in the aqueous phase. To date, researchers have not applied the ultrafine shearing cell disruption method in the extraction of C-PC. 

A novel ultrafine shearing cell disruption method is applied to develop a viable and economical extraction method for large-scale C-PC production. This study carried out five different cell disruption methods (soaking, ultrasonication, freezing-thawing, soaking-ultrafine shearing and soaking-ultrafine shearing-ultrasonication) to disrupt *S. platensis* cells and extract C-PC. The yields of C-PC from the different cell disruption methods were compared. Finally, *S. platensis* was confirmed to be the most efficient cell disruption method.

## 2. Results and Discussion

### 2.1. Effect of Soaking Time on the Yield of C-PC

**Figure 1:**
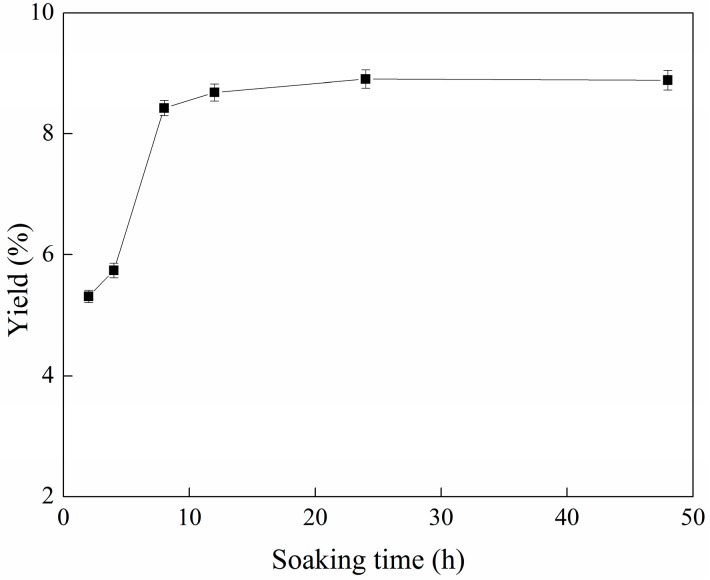
Relationship between soaking time and yield of C-PC. Error bars indicate standard deviations of the means (*n* = 3).

Figure 1 shows the influence of the soaking time on the yield of C-PC, with the soaking time ranging from 2 h to 48 h.

It can be seen from Figure 1 that, with the increase of the soaking time, the yield of C-PC first increased, and then gradually stabilized. When the soaking time was 24 h, the yield of C-PC reached 8.91%, and when the soaking time was 48 h, the yield of C-PC reached 8.89%, which was close to the stability of the phycocyanin yield. The comprehensive analysis showed that the soaking time range from 1 h to 24 h had a positive effect on the cell disruption of *S. platensis*, and it had a negligible effect on the yield of C-PC after 24 h of soaking, because protein denaturation had occurred. Considering the cycle time of industrial production, the most suitable soaking time was concluded to be 8–12 h.

### 2.2. Effect of Ultrafine Shearing Time on the Yield of C-PC

**Figure 2:**
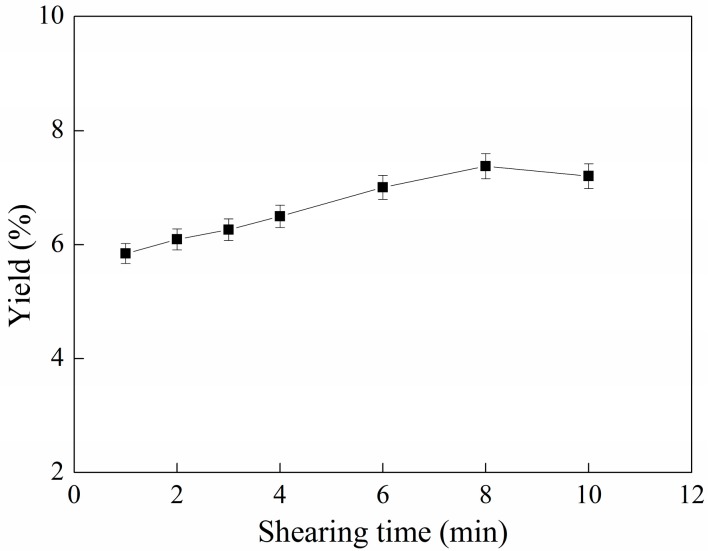
Relationship between shearing time and yield of C-PC. Error bars indicate standard deviations of the means (*n* = 3).

The influence of the ultrafine shearing time on the yield of C-PC is shown in Figure 2.

Figure 2 illustrates that, with the increase of the shearing time, the yield of phycocyanin increased; the yield of phycocyanin first increased, and then gradually stabilized. With a longer shearing time, the possibility of disruption of the cells of *S. platensis* is higher. When the shearing time was 8 min, the yield of phycocyanin reached a maximum of 7.37%. The suspended cells may have been disrupted in the shearing zones by shear forces with energy transfer from blades to the cells. The protein leaked from the disrupted cells under the shearing force. After 8 min, the curve appeared to slightly decrease. This may be due to the heat generated by the mechanical power leading to a small part of the protein being damaged. It can be seen that the ultrafine shearing time has a positive effect on the wall-breaking of algal blue cells, and the lowest yield value of 5.84% was obtained when the ultrafine shearing time was 1 min. However, after only 6–8 min of shearing operation, the yield had increased to 7.37%. The yield increase was relatively large in a short few minutes. In summary, the ultrafine shearing method is an efficient method for cell disruption, and it has definite potential value in industrial production.

### 2.3. Effect of Ultrasonication Time on the Yield of C-PC

**Figure 3:**
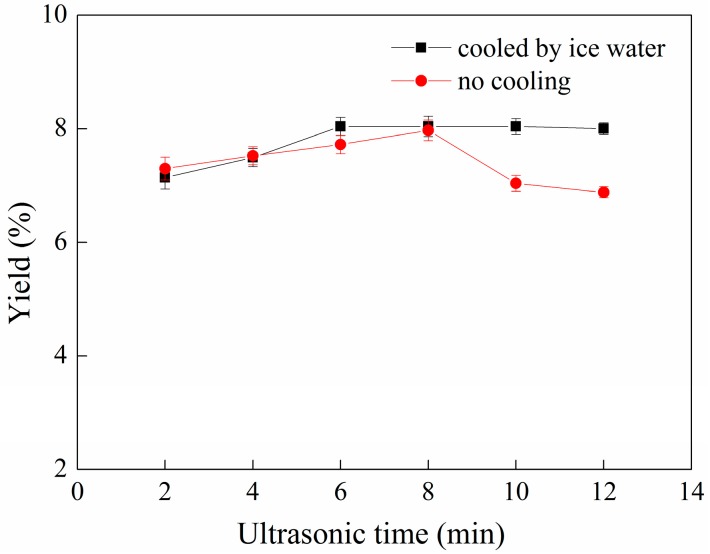
Relationship between ultrasonication time and yield of C-PC. Error bars indicate standard deviations of the means (*n* = 3).

As mentioned earlier, maceration of dried powder for 4 h resulted in a C-PC yield of 5.74%. Ultrasonication can cause cavitation between solvent and samples and can disrupt the cell wall [20]. During extraction of phycobiliproteins from macroalgae, Mittal, Tavanandi, Mantri and Raghavarao [21] confirmed that ‘maceration + ultrasonication’ resulted in higher extraction efficiency compared to maceration alone or ultrasonication alone. The sample was soaked for 4 h, and then treated by ultrasonication for different times. The influence of the ultrasonication time on the yield of C-PC is shown in Figure 3. Ultrasounds enhance mass transport by disrupting cell walls [22]. The greater the ultrasonic energy applied, the more the cavitation effect is happened; and thus, the greater the volume of C-phycocyanin that can be extracted from *Spirulina platensis*.

From Figure 3, the effect of the ultrasonication time on the yield of C-PC can be observed. When the sample was treated without the cooling condition, the yield of C-PC reached 7.14% with an ultrasonication time of 2 min. When the curve reached the maximum point (8 min ultrasonication time), the yield was 7.97%; then, the yield decreased sharply. The main reason for the change is probably the high temperature generated by the ultrasound, which results in damage to the dissociated protein. Ultrasonic disruption can generate considerable heat with longer ultrasonication times [23]. An ultrasonication time that is too long will result in the temperature of the testing sample reaching over 35 °C, which is not desirable for C-PC. After 8 min of ultrasonication time, the heat produced by ultrasound becomes the main influencing factor, and the curve drops sharply.

The second case is when the sample was cooled by ice water during ultrasonication. Under this condition, the yield of C-PC reached 8.04%, and then became constant. A longer ultrasonication time does not always result in a desirable cell disruption [24]. In summary, the ultrasonication method can disrupt the cell wall, but the heat generated during the ultrasonication process has great influence on the yield of phycocyanin. Hence, the ultrasonication time should be controlled to within a certain range to avoid overheating.

### 2.4. Effect of Cycles of Freezing-Thawing on the Yield of C-PC

**Figure 4:**
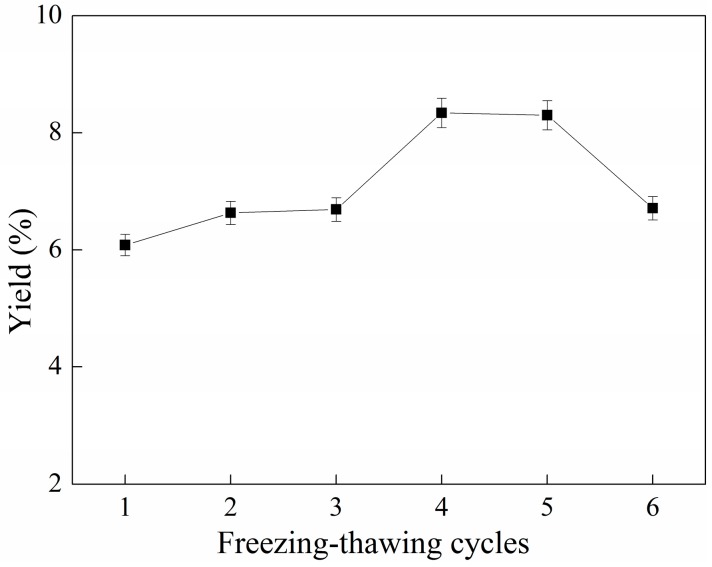
Relationship between freezing-thawing cycles and yield of C-PC. Error bars indicate standard deviations of the means (*n* = 3).

The freezing-thawing method is predominantly applied in the laboratory-scale extraction of C-PC. Generally, the cell suspension is frozen (at −20 °C) and thawed (at 25 °C) repeatedly, four or five times [25,26]. The influence of the cycles of freezing-thawing on the yield of phycocyanin is shown in Figure 4.

In Figure 4, the yield of phycocyanin continuously increases with the increase of freezing-thawing cycles when the cycle number is below 4. After four cycles of freezing and thawing, the effect of cell wall rupture was the best, and the yield of C-PC reached 8.26%. It can be seen from the curve that, with freezing and thawing for 1–3 times, there is little change in C-PC extraction. When freezing and thawing for four cycles, the yield of phycocyanin increased significantly. Wang, Liu, Ma, Cui and Shao [26] conducted cell disruption of *S. platensis* by freezing-thawing repeatedly for four cycles, obtaining a concentration of C-PC of 0.46 mg/mL. When freezing and thawing for five cycles, the yield of phycocyanin decreased slightly. This may be due to repeated and rapid temperature changes, leading to protein denaturation. The freezing and thawing method of disruption of the Spirulina cell wall requires an ultra-low-temperature environment, so this method is heavily energy-consuming in large-scale industrial production.

### 2.5. Effect of Soaking Time and Ultrafine Shearing Time on the Yield of C-PC

**Figure 5:**
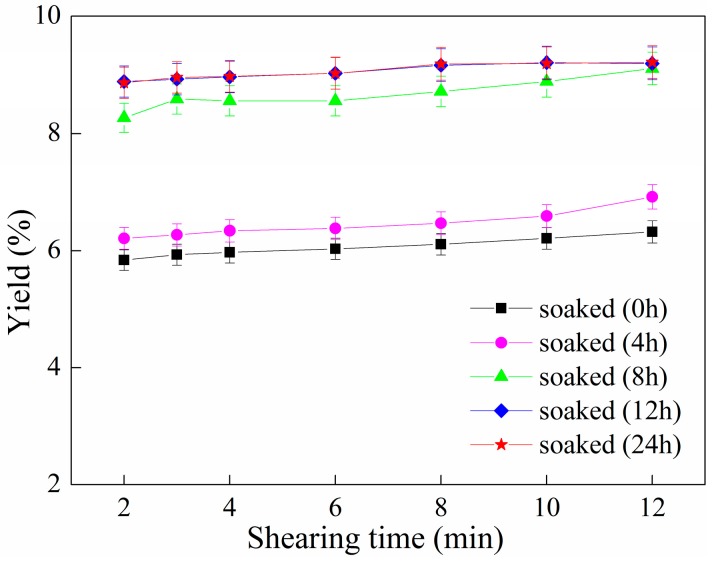
Relationship of soaking time and ultrafine shearing time on yield of C-PC. Error bars indicate standard deviations of the means (*n* = 3).

The influence of the soaking time and ultrafine shearing time on the yield of phycocyanin is shown in Figure 5.

In Figure 5, under the same shearing time, when the soaking time was increased from 0 h to 12 h, the yield of phycocyanin increased. In addition, when the soaking time and ultrafine shearing time were fixed at 8 h and 8 min, respectively, the yield of phycocyanin reached 8.89%, which was close to the yield of 9.2% achieved with soaking times of 12 h and 24 h. With soaking times of 12 h or 24 h, the yield of phycocyanin was not improved with different shearing times. After 12 h of soaking, the phycocyanin in the Spirulina has been sufficiently released. Based on the above analysis, the ultrafine shearing method is able to greatly shorten the soaking time to 4 hours, thereby reducing the extraction time. This has practical significance for industrial production.

### 2.6. The Combined Effect of the Soaking Method, Ultrafine Shearing Method and Ultrasonication Method on the Yield of C-PC

**Figure 6:**
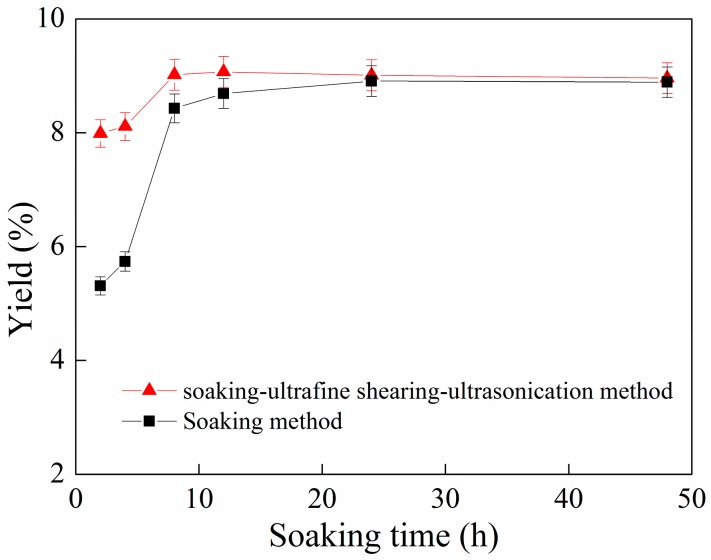
Relationship of soaking time and yield of C-PC based on the method of soaking-ultrafine shearing-ultrasonication. Error bars indicate standard deviations of the means (*n* = 3).

The influence of the soaking-ultrafine shearing-ultrasonication method on the yield of phycocyanin is shown in Figure 6.

From Figure 6, it can be seen that the yield of phycocyanin increases gradually with soaking time in the case of both the soaking method alone and the soaking-ultrafine shearing-ultrasonication method. For the soaking method alone, the yield of C-PC was 8.43%, 8.69% and 8.91% for soaking times of 8 h, 12 h and 24 h, respectively. For the soaking-ultrafine shearing-ultrasonication method, the yield of C-PC was 8.11%, 9.02% and 9.07% for soaking times of 8 h, 12 h and 24 h, respectively, when the shearing time was fixed at 8 min and the ultrasonication time was fixed at 10 min. According to the above analysis, it can be concluded that the effect of the soaking-ultrafine shearing-ultrasonication method on Spirulina cell disruption is better under the current experimental conditions.

### 2.7. The Combined Effect of the Soaking Method, Ultrafine Shearing Method and Ultrasonication Method on the Yield of C-PC

**Figure 7:**
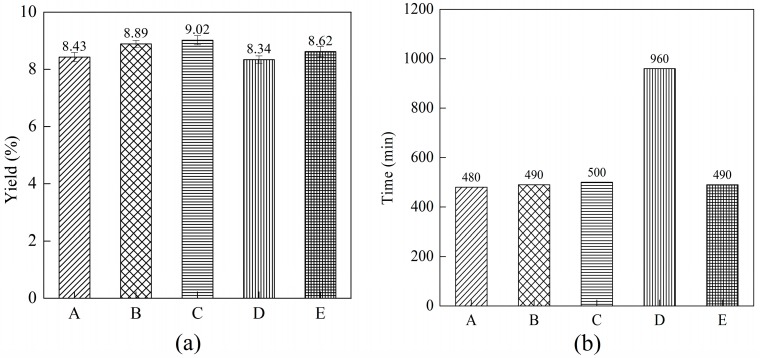
Comparison of the different cell disruption methods. Error bars indicate standard deviations of the means (*n* = 3): (**a**) the yield of C-PC; (**b**) the extraction time.

The comparison of the different cell disruption methods on the yield of phycocyanin is shown in Figure 7.

In Figure 7a, A represents the soaking method, B represents the soaking-ultrafine shearing method, C represents the soaking-ultrafine shearing-ultrasonication method, D represents the freezing-thawing method and E represents the ultrasonication method. With the soaking time at 8 h, the shearing time at 10 min and the ultrasonication time at 10 min, the yield of C-PC was 8.43%, 8.89%, 9.02%, 8.34%, and 8.62% by using the soaking, soaking-ultrafine shearing, soaking-ultrafine shearing-ultrasonication, freezing-thawing (4 cycles) and ultrasonication methods, respectively. The energy consumption levels for methods A, B, C, D, E and F were 0 W, 40.92 W, 60.4 W, 233 W and 19.48 W, respectively. Figure 7a demonstrates that the yield of C-PC is highest when using the soaking-ultrafine shearing-ultrasonication method. Compared with the soaking method, the ultrasonication method was able to improve the yield of C-PC under the conditions of 8 h of soaking time and 10 min of ultrasonication time. Meanwhile, under the conditions of 8 h of soaking time and 10 min of ultrafine shearing time, the yield of the C-PC was able to be improved significantly. It can also be seen from Figure 7a that the yield of C-PC obtained by the freezing-thawing method was 8.34%, which is relatively high, but this method consumes a great deal of time and energy. There were significant differences (*p* < 0.0001) in the yield of C-PC among the five cell disruption methods. Meanwhile, the yield of C-PC was statistically significant (*p* < 0.0001) between the soaking-ultrafine shearing method and the soaking-ultrafine shearing-ultrasonication method. In Figure 7b, the extraction time under the five different treatment method was compared. The freezing-thawing method needs the longest extraction time of the five methods. For the other four extraction methods, the extraction time was similar.

**Figure 8:**
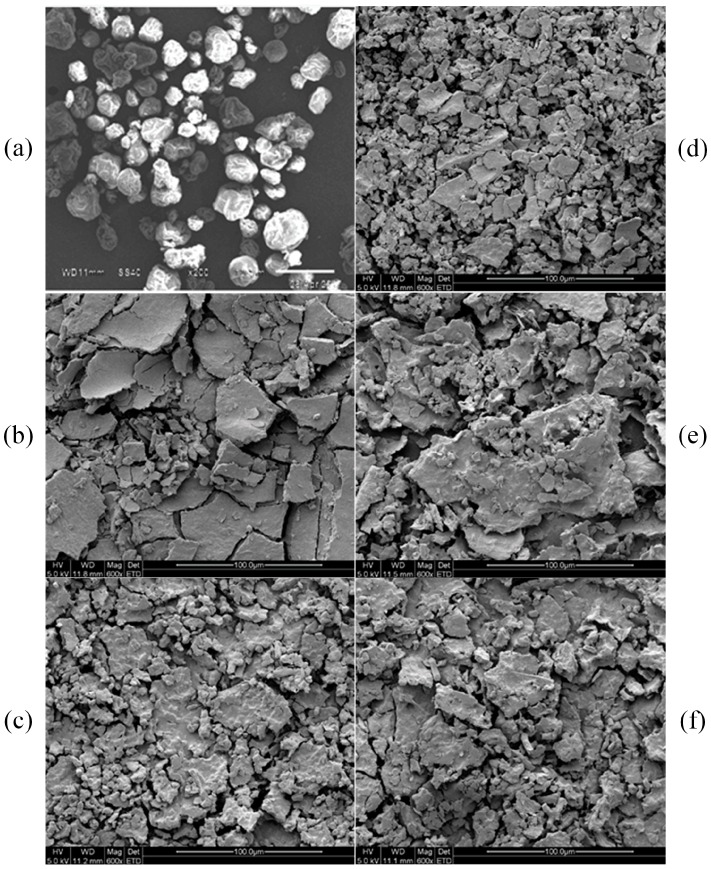
Scanning electron micrographs of cell surfaces: (**a**) Cells before treatment; (**b**) cells with soaking treatment; (**c**) cells with soaking-ultrafine shearing treatment; (**d**) cells with soaking-ultrafine shearing-ultrasonication treatment; (**e**) cell with freezing-thawing treatment; (**f**) cell with ultrasonication treatment.

Scanning electron micrographs of six samples are shown in Figure 8. Figure 8a exhibits the intact cellular wall before treatment. In this case, the initial cell structure of *S. platensis* shows the cell walls are smooth and intact. Figure 8b shows that the cell wall of *S. platensis* was partially ruptured after the soaking treatment. The ruptured condition of the cellular wall and membranes can be observed in Figure 8c. From Figure 8c, we can observe that soaking-ultrafine shearing treatment had a better effect than soaking on the rupture of cellular tissue. Figure 8d shows the microstructure of ruptured tissue after treatment by soaking-ultrafine shearing-ultrasonication. From Figure 8d, it can be seen that the cell structures were disrupted thoroughly, and C-phycocyanin can be thoroughly extracted using the soaking-ultrafine shearing-ultrasonication method. Figure 8e shows the microstructure of ruptured tissue after treatment by freezing-thawing method. Figure 8f shows the microstructure of ruptured tissue after treatment by ultrasonication method. In Figure 8f, it can be clearly observed that high deformation occurred in the cell wall, this result confirms the high yield achieved with this mechanical disruption technique.

In a word, the effect of the soaking-ultrafine shearing-ultrasonication method on the disruption of Spirulina cells is more obvious. The soaking-ultrafine shearing-ultrasonication is now being applied for the first time in the industrial-scale extraction process of C-PC from *Spirulina platensis* in the Dongtai City Spirulina Bio-engineering Co., Ltd. (Dongtai City, Jiangsu, China), where it facilitates and promotes extraction efficiency and saves energy consumption.

## 3. Materials and Methods 

### 3.1. Materials

Dried *S. platensis* powder was supplied by Heyi Biotechnology Co., Ltd. (Xi’an, China). Ammonium sulphate (analytical grade) was produced by Zhiyuan Chemical Reagent Co., Ltd. (Tianjin, China). Potassium dihydrogen phosphate (analytical grade), hydrochloric acid (analytical grade), etc. were supplied by Sinopharm Chemical Reagent Co., Ltd. (Shanghai, China). Deionized water was supplied by Logistic Division, Jiangnan University (Jiangsu, China).

The powder was weighed by an analytical balance (±0.1 mg) (AR 1140, Ohaus, Parsippany, NJ, USA). The testing sample was prepared by mixing a certain weight of *S. platensis* powder with a volume of phosphate buffer (pH 6.5). The solvent-to-solid ratio was equal to 20 mL/g. The mixture was stored at 4 °C in a Bunsen beaker for further cell disruption. After the extraction, the solution of the resulting extract was transferred to a centrifuge tube for centrifugation at 15,000 rpm for 15 min by a high-speed centrifuge (TGL-16C, Anting Scientific Instrument Company, Shanghai, China) to remove cell debris. After centrifugation, the supernatants were collected and stored at 4 °C for further analysis.

### 3.2. Methods

#### 3.2.1. Concentration and Yield of C-PC

The concentration of C-PC was calculated according to the method described by Bennett and Bogorad [27]:

P_c_ = (A_620_ − 0.474A_652_)/5.34, (1)

The yield of C-PC was calculated as follows:

Y(%) = (P_c_ × V × n)/(D_B_ × 1000) × 100%, (2)

where P_c_ (mg/mL) is the concentration of C-PC, Y is the yield of C-PC, A_620_ is the absorbance of the sample at a wavelength of 620 nm, A_652_ is the absorbance of the sample at a wavelength of 652 nm, V (mL) is the volume of the crude phycocyanin extract, n is the dilution factor and D_B_ (g) is the dry weight of *S. platensis* powder.

#### 3.2.2. Soaking

Six parts of the test samples were soaked in the phosphate buffer (pH 6.5) for 2, 4, 8, 12, 24 and 48 h in an electrothermal constant temperature water tank (model: DK-80, Sanfa Scientific Instrument Co., Ltd., Shanghai, China). Each solution was centrifuged (15 min, 15,000 rpm) by using a high-speed centrifuge (model: TGL-16C, Anting Scientific Instrument Factory, Shanghai, China). After centrifugation, we took the supernatant and added a volume of 20% ammonium sulphate aqueous solution for salting-out. The precipitate was removed via centrifugation of the salting-out aqueous solution and dissolved in phosphate buffer aqueous solution to obtain the final phycocyanin aqueous solution. The absorbance of the C-PC was measured by an ultraviolet spectrophotometer (UV-1800, Shimadzu, Kyoto, Japan) at room temperature, and then the concentration and yield of the C-PC were calculated according to Equation (1).

#### 3.2.3. Ultrasonication

After 4 h of soaking at low temperature (at 4 °C), the soaking solution was divided into 6 aliquots and treated with an ultrasonic cell grinder (model: JY99-IIDN, New Chi Technology Co., Ltd., Ningbo, China) for 2, 4, 6, 8, 10 and 12 min. The ultrasonic cell grinder had a working power fixed at 90 W. Because the overheating produced by ultrasonic treatment may cause protein denaturation, the sample baker was placed in ice water to absorb the ultrasonication heat during treatment. Meanwhile, the ultrasonic cell grinder was operated with an intermittent working mode (2 s on, 5 s off).

#### 3.2.4. Freezing-Thawing

The freezing-thawing method consists of freezing and thawing of *Spirulina* solution repeatedly. Because of its mild conditions for cell disruption, the protein is not easily damaged. When frozen, small ice particles form in the cell. Pressure on the cell wall is exerted by freezing the intracellular fluids, as well as the extraction buffer. Then, the cracked cells absorb water and burst, releasing protein into the extraction buffer. 

The test sample was frozen at −20 °C for 4 h. The frozen solution was removed and subjected to a 25 °C water bath treatment for 20 min. One fifth of the solution was centrifuged (15 min, 15,000 rpm) and the remaining solution was subjected to freezing-thawing for a total of 6 cycles. The suspension was subjected to centrifugation, and the supernatant was collected for further analysis.

#### 3.2.5. Soaking-Ultrafine Shearing

Five parts of the test samples were subjected to soaking for 0, 4, 8, 12 and 24 h. Each solution was divided into 7 equal portions, and the aliquots were treated with an ultrafine food shearing machine for 2, 3, 4, 6, 8, 10 and 12 min.

#### 3.2.6. Soaking-Ultrafine Shearing-Ultrasonication

After 2, 4, 8, 12, 24 and 48 h of soaking, each solution was extracted, and the aliquots were treated with an ultrafine food shearing machine for 10 min. The shearing solution was treated with an ultrasonic cell grinder for 10 min and then centrifuged at 15,000 rpm for 15 min. Last, the supernatant was extracted.

#### 3.2.7. Scanning Electron Micrograph (SEM) Analysis 

Six types of sample were prepared for a better visualization of the degree of internal cell disruption. One was initial powder that had not been treated by any extraction method, and the other five samples were treated by soaking, soaking-ultrafine shearing, soaking-ultrafine shearing-ultrasonication, freezing-thawing and ultrasonication, respectively.

Finally, a scanning electron microscope (Quanta-200, FEI Company, Hillsboro, OR, USA) with an accelerating voltage of 50 kV was applied for observing the microscopic changes of the cell surfaces.

#### 3.2.8. Statistical Analysis

One-way ANOVA was used to examine the effect of cell disruption of the five methods. Moreover, the post hoc test (by one-way ANOVA) was executed to distinguish the differences in the soaking-ultrafine shearing method and the soaking-ultrafine shearing-ultrasonication method. The significance level (a) was set to 0.05. All data processing, data management, and statistical analysis was performed using SAS version 9.3 (SAS Institute Inc., Cary, NC, USA). All the experiments were performed with 3 replicates.

## 4. Conclusions

The ultrafine shearing method is first applied for the cell disruption of *S. platensis*. The ultrafine shearing method combined with soaking and ultrasonication can shorten the extraction time and obtain a higher yield of C-PC compared to the conventional soaking method. Under the conditions of a soaking time of 8 h, a shearing time of 10 min, and an ultrasonication time of 10 min, the yield of phycocyanin reached 9.02%. As a novel type of cell disruption technology, the soaking-ultrafine shearing-ultrasonication method has the potential to be applied in the large-scale extraction of C-PC from *S. platensis*. The findings of this research provide a valuable means for saving time and energy in the extraction of C-PC.
